# Supplementary material for: Evolutionary recruitment of flexible Esrp-dependent splicing programs into diverse embryonic morphogenetic processes
Source: Nat Commun. 2017 Nov 27;8:1799. doi: 10.1038/s41467-017-01961-y (PMC5703972; doi:10.1038/s41467-017-01961-y)
Supplement: Supplementary file 1 — Supplementary Information [file 41467_2017_1961_MOESM1_ESM.pdf]

Supplementary Materials for:

Evolutionary recruitment of flexible *Esrp*-dependent splicing programs into diverse embryonic morphogenetic processes

Demian Burguera, Yamile Marquez, Claudia Racioppi, Jon Permanyer, Antonio Torres, Rosaria Esposito, Beatriz Albuixech-Crespo, Lucía Fanlo, Ylenia D'Agostino, Andre Gohr, Enrique Navas-Perez, Ana Riesgo, Claudia Cuomo, Giovanna Benvenuto, Lionel A. Christiaen, Elisa Martí, Salvatore D'Aniello, Antonietta Spagnuolo, Filomena Ristoratore, Maria Ina Arnone, Jordi Garcia-Fernàndez, Manuel Irimia

Supplementary Table 1. This table contains the 22 clusters of homologous exons that were detected as *Esrp*-dependent in zebrafish, human and mouse. ESRP_DEP-SIL, *Esrp*-dependent exon upregulated upon *Esrp* KD; ESRP_DEP-ENH, *Esrp*-dependent exon downregulated upon *Esrp* KD; NR, non-*Esrp* regulated; NC, not sufficient read coverage to estimate changes in PSI; AS, alternatively spliced (PSI between 10 and 90); HIGH_PSI, constitutively spliced (PSI higher than 90); NI, no information available.

Cluster_ID	Species	Gene ID	Gene name	Exon genomic coordinates	Exon size	Vast-tools ID	ESRP_ DEP	Exon type
CL010896.01	Dre	ENSDARG00000078675	dock7	chr6:32379767-32379859	93	DreEX0028016	ESRP_DEP-SIL	AS
CL010896.01	Hsa	ENSG00000116641	DOCK7	chr1:63010618-63010710	93	HsaEX0020639	ESRP_DEP-SIL	AS
CL010896.01	Mmu	ENSMUSG00000028556	Dock7	chr4:98660338-98660427	90	MmuEX0015458	ESRP_DEP-SIL	AS
CL004509.01	Dre	ENSDARG00000010155	abi1a	chr24:5976265-5976351	87	DreEX0010307	NR	AS
CL004509.01	Dre	ENSDARG00000014875	abi2b	chr6:9770304-9770390	87	DreEX0010332	ESRP_DEP-SIL	AS
CL004509.01	Hsa	ENSG00000138443	ABI2	chr2:204276008-204276094	87	HsaEX0000647	NR	AS
CL004509.01	Hsa	ENSG00000136754	ABI1	chr10:27044584-27044670	87	HsaEX0000644	ESRP_DEP-SIL	AS
CL004509.01	Mmu	ENSMUSG00000058835	Abi1	chr2:22805704-22805790	87	MmuEX0003244	ESRP_DEP-SIL	AS
CL010468.01	Dre	ENSDARG00000020488	arhgap17a	chr12:20352804-20353067	264	DreEX0014246	ESRP_DEP-SIL	AS
CL010468.01	Hsa	ENSG00000140750	ARHGAP17	chr16:24950685-24950918	234	HsaEX0005421	ESRP_DEP-SIL	AS
CL010468.01	Mmu	ENSMUSG00000030766	Arhgap17	chr7:130437986-130438219	234	MmuEX0005800	ESRP_DEP-SIL	AS
CL016338.02	Dre	ENSDARG00000078233	ctnnd1	chr1:43494933-43495084	152	NA	ESRP_DEP-SIL	AS
CL016338.02	Hsa	ENSG00000198561	CTNND1	chr11:57558857-57559145	289	HsaEX0017869	ESRP_DEP-SIL	AS
CL016338.02	Mmu	ENSMUSG00000034101	Ctnnd1	chr2:84464427-84464715	289	MmuEX0013073	ESRP_DEP-SIL	AS
CL014001.01	Dre	ENSDARG00000012078	meis1b	chr13:5367347-5367440	94	DreEX0045964	ESRP_DEP-SIL	AS
CL014001.01	Hsa	ENSG00000143995	MEIS1	chr2:66796182-66796277	96	HsaEX0038792	ESRP_DEP-SIL	AS
CL014001.01	Mmu	ENSMUSG00000020160	Meis1	chr11:18783991-18784085	95	MmuEX0028623	ESRP_DEP-SIL	AS
CL008378.29	Dre	ENSDARG00000009499	syne1a	chr20:26450144-26450212	69	DreEX0076807	ESRP_DEP-SIL	AS
CL008378.29	Hsa	ENSG00000131018	SYNE1	chr6:152466622-152466690	69	HsaEX0063158	ESRP_DEP-SIL	AS

CL008378.29	Mmu	ENSMUSG00000019769	Syne1	chr10:5301207-5301275	69	MmuEX0045949	ESRP_DEP-SIL	AS
CL012712.01	Dre	ENSDARG00000011027	fgfr1a	chr8:51473548-51473695	148	DreEX0032641	ESRP_DEP-ENH	AS
CL012712.01	Dre	ENSDARG00000011027	fgfr1a	chr8:51468017-51468161	145	DreEX0032642	ESRP_DEP-SIL	AS
CL012712.01	Dre	ENSDARG00000011190	fgfr1b	chr10:42810564-42810714	151	DreEX0032649	LC	AS
CL012712.01	Dre	ENSDARG00000011190	fgfr1b	chr10:42811058-42811202	145	NA	LC	AS
CL012712.01	Dre	ENSDARG00000004782	fgfr3	chr13:13108015-13108159	145	DreEX0032695	LC	AS
CL012712.01	Dre	ENSDARG00000004782	fgfr3	chr13:13106931-13107075	145	DreEX0032696	ESRP_DEP-SIL	AS
CL012712.01	Dre	ENSDARG00000058115	fgfr2	chr13:46214695-46214839	145	DreEX0032684	ESRP_DEP-SIL	AS
CL012712.01	Dre	ENSDARG00000058115	fgfr2	chr13:46216912-46217050	139	DreEX0032683	ESRP_DEP-ENH	AS
CL012712.01	Hsa	ENSG00000066468	FGFR2	chr10:123278196-123278343	148	HsaEX0025678	ESRP_DEP-ENH	AS
CL012712.01	Hsa	ENSG00000066468	FGFR2	chr10:123276833-123276977	145	HsaEX0025679	ESRP_DEP-SIL	AS
CL012712.01	Hsa	ENSG00000068078	FGFR3	chr4:1804641-1804791	151	HsaEX0025686	ESRP_DEP-SIL	AS
CL012712.01	Hsa	ENSG00000068078	FGFR3	chr4:1805419-1805563	145	HsaEX0025687	ESRP_DEP-SIL	AS
CL012712.01	Hsa	ENSG00000077782	FGFR1	chr8:38280543-38280693	151	HsaEX0025665	ESRP_DEP-ENH	AS
CL012712.01	Hsa	ENSG00000077782	FGFR1	chr8:38279315-38279459	145	NA	LC	AS
CL012712.01	Mmu	ENSMUSG00000030849	Fgfr2	chr7:137343272-137343419	148	NA	ESRP_DEP-ENH	AS
CL012712.01	Mmu	ENSMUSG00000030849	Fgfr2	chr7:137341933-137342077	145	MmuEX0019159	ESRP_DEP-SIL	AS
CL012712.01	Mmu	ENSMUSG00000054252	Fgfr3	chr5:34073930-34074080	151	MmuEX0019169	ESRP_DEP-ENH	AS
CL012712.01	Mmu	ENSMUSG00000054252	Fgfr3	chr5:34074766-34074910	145	MmuEX0019170	ESRP_DEP-SIL	AS
CL012712.01	Mmu	ENSMUSG00000031565	Fgfr1	chr8:26673846-26673996	151	NA	ESRP_DEP-ENH	AS
CL012712.01	Mmu	ENSMUSG00000031565	Fgfr1	chr8:26674812-26674956	145	NA	ESRP_DEP-SIL	HIGH_PSI
CL012712.01	Spu	WHL22.323968	Fgfr=FGFR1	Scaffold2483:151430-151571	142	SpuEX0011658	NR	HIGH_PSI
CL012712.02	Dre	ENSDARG00000058115	fgfr2	chr13:46250743-46251009	267	DreEX0032685	ESRP_DEP-SIL	AS
CL012712.02	Hsa	ENSG00000066468	FGFR2	chr10:123324952-123325218	267	HsaEX0025680	ESRP_DEP-SIL	AS
CL012712.02	Mmu	ENSMUSG00000030849	Fgfr2	chr7:137385892-137386158	267	MmuEX0019154	ESRP_DEP-SIL	AS
CL011475.01	Dre	ENSDARG00000061525	slka	chr13:24666762-24666854	93	NA	ESRP_DEP-ENH	AS
CL011475.01	Hsa	ENSG00000065613	SLK	chr10:105770574-105770666	93	HsaEX0060174	ESRP_DEP-ENH	AS
CL011475.01	Mmu	ENSMUSG00000025060	Slk	chr19:47702138-47702230	93	MmuEX0043706	ESRP_DEP-ENH	AS

CL002719.01	Dre	ENSDARG00000069946	itga6b	chr1:30258429-30258522	94	DreEX0040093	ESRP_DEP-ENH	AS
CL002719.01	Dre	ENSDARG00000042282	itga6a	chr9:3511546-3511644	99	NA	NC	NI
CL002719.01	Hsa	ENSG00000091409	ITGA6	chr2:173366500-173366629	130	HsaEX0032370	ESRP_DEP-ENH	AS
CL002719.01	Mmu	ENSMUSG00000027111	Itga6	chr2:71691591-71691720	130	MmuEX0024617	ESRP_DEP-ENH	AS
CL003118.01	Dre	ENSDARG00000103125	TSC2	chr1:53549954-53550082	129	DreEX0009092	ESRP_DEP-ENH	AS
CL003118.01	Hsa	ENSG00000103197	TSC2	chr16:2127599-2127727	129	HsaEX0067508	ESRP_DEP-ENH	AS
CL003118.01	Mmu	ENSMUSG00000002496	Tsc2	chr17:24743169-24743297	129	MmuEX0049266	ESRP_DEP-ENH	AS
CL000259.01	Dre	ENSDARG00000020469	map3k7	chr20:24266265-24266345	81	DreEX0044679	ESRP_DEP-ENH	AS
CL000259.01	Hsa	ENSG00000135341	MAP3K7	chr6:91254271-91254351	81	HsaEX0037602	ESRP_DEP-ENH	AS
CL000259.01	Mmu	ENSMUSG00000028284	Map3k7	chr4:32081849-32081929	81	MmuEX0027748	ESRP_DEP-ENH	AS
CL009534.02	Dre	ENSDARG00000005754	ptprfb	chr2:18006258-18006284	27	DreEX0059640	ESRP_DEP-ENH	AS
CL009534.02	Dre	ENSDARG00000104937	CABZ01084323.1	KN150000.1:26209-26235	27	NA	NC	NI
CL009534.02	Hsa	ENSG00000142949	PTPRF	chr1:44067742-44067768	27	HsaEX0051138	ESRP_DEP-ENH	AS
CL009534.02	Mmu	ENSMUSG00000033295	Ptprf	chr4:117900424-117900450	27	MmuEX0037910	ESRP_DEP-ENH	AS
CL013859.01	Dre	ENSDARG00000104279	appa	chr1:638055-638222	168	DreEX0013999	ESRP_DEP-ENH	AS
CL013859.01	Hsa	ENSG00000084234	APLP2	chr11:129993507-129993674	168	HsaEX0005149	ESRP_DEP-ENH	AS
CL013859.01	Mmu	ENSMUSG00000031996	Aplp2	chr9:30975146-30975313	168	MmuEX0005570	ESRP_DEP-ENH	AS
CL003579.01	Dre	ENSDARG00000027279	numb	chr17:51739829-51739981	153	DreEX0052085	ESRP_DEP-ENH	AS
CL003579.01	Hsa	ENSG00000133961	NUMB	chr14:73745989-73746132	144	HsaEX0044216	ESRP_DEP-ENH	AS
CL003579.01	Mmu	ENSMUSG00000021224	Numb	chr12:85138147-85138293	147	MmuEX0032646	ESRP_DEP-ENH	AS
CL010697.01	Dre	ENSDARG00000019581	uso1	chr1:25797430-25797450	21	DreEX0083646	ESRP_DEP-ENH	AS
CL010697.01	Hsa	ENSG00000138768	USO1	chr4:76716489-76716509	21	HsaEX0069561	ESRP_DEP-ENH	AS
CL010697.01	Mmu	ENSMUSG00000029407	Uso1	chr5:92612508-92612528	21	MmuEX0050799	ESRP_DEP-ENH	AS
CL001791.04	Dre	ENSDARG00000098374	FLNB (2 of 2)	chr11:41969117-41969200	84	DreEX0004329	ESRP_DEP-ENH	AS
CL001791.04	Hsa	ENSG00000136068	FLNB	chr3:58127585-58127656	72	HsaEX0025930	ESRP_DEP-ENH	AS

CL001791.04	Mmu	ENSMUSG00000025278	Flnb	chr14:8755189-8755260	72	NA	ESRP_DEP-ENH	AS
CL014228.02	Dre	ENSDARG00000019345	mark3a	chr13:15527303-15527329	27	DreEX0045082	NR	AS
CL014228.02	Dre	ENSDARG00000026630	mark3b	chr20:43906181-43906207	27	DreEX0045098	NR	AS
CL014228.02	Dre	ENSDARG00000079905	mark2a	chr21:27174765-27174791	27	DreEX0045060	ESRP_DEP-ENH	AS
CL014228.02	Hsa	ENSG00000072518	MARK2	chr11:63673560-63673586	27	HsaEX0037916	ESRP_DEP-ENH	AS
CL014228.02	Mmu	ENSMUSG00000024969	Mark2	chr19:7354476-7354502	27	MmuEX0027951	ESRP_DEP-ENH	AS
CL002302.01	Dre	ENSDARG00000052170	uap1	chr6:35056027-35056068	42	DreEX0082619	ESRP_DEP-ENH	AS
CL002302.01	Hsa	ENSG00000117143	UAP1	chr1:162562522-162562572	51	HsaEX0068590	ESRP_DEP-ENH	AS
CL002302.01	Mmu	ENSMUSG00000026670	Uap1	chr1:172078127-172078177	51	MmuEX0050061	ESRP_DEP-ENH	AS
CL002302.01	Spu	WHL22.63642	Udp1=UAP1	Scaffold1209:59560-59595	36	SpuEX0043794	NR	AS
CL010989.01	Dre	ENSDARG00000027867	paplna	chr17:51080927-51080998	72	DreEX0053392	ESRP_DEP-ENH	AS
CL010989.01	Hsa	ENSG00000100767	PAPLN	chr14:73719869-73719934	66	HsaEX0045433	ESRP_DEP-ENH	AS
CL010989.01	Mmu	ENSMUSG00000021223	Papln	chr12:85116799-85116864	66	MmuEX0033595	ESRP_DEP-ENH	AS
CL015350.02	Dre	ENSDARG00000032114	slain2	chr20:23551888-23551968	81	DreEX0071877	ESRP_DEP-ENH	AS
CL015350.02	Hsa	ENSG00000109171	SLAIN2	chr4:48396593-48396670	78	HsaEX0058524	ESRP_DEP-ENH	AS
CL015350.02	Mmu	ENSMUSG00000036087	Slain2	chr5:73357043-73357120	78	MmuEX0042523	ESRP_DEP-ENH	AS
CL010608.03	Dre	ENSDARG00000078441	ppip5k2	chr10:6857946-6858119	174	DreEX0057483	ESRP_DEP-ENH	AS
CL010608.03	Hsa	ENSG00000145725	HISPPD1	chr5:102518935-102519108	174	HsaEX0030034	ESRP_DEP-ENH	AS
CL010608.03	Mmu	ENSMUSG00000040648	Ppip5k2	chr1:99617683-99617856	174	MmuEX0036612	ESRP_DEP-ENH	AS

Supplementary Table 2. This table contains all primer sequences used in this study.

DreESRP1_probe_Fw	GTCACCTGGATTTGATCTCT
DreESRP1_probe_Rv	ATCTGATTCTCCCTTGACAG
DreESRP2_probe_Fw	GATTGCAGGAGGCACTTCCA
DreESRP2_probe_Rv	AGCTCATATTCATGTTCATG
Dre_exon1_gESRP1_Fw	TAGGAGCAAGTGGGGATAAGTT
Dre_exon1_gESRP1_Rv	AAACAACTTATCCCCACTTGC
DreEsrp1_genotype_Fw	GTCACCTGGATTTGATCTCT
DreEsrp1_genotype_Rv	TCAAATTGATATGTGTAGCC
Dre_exon3_gESRP2_Fw	TAGGAGACCGGGCTCACTGCCG
Dre_exon3_gESRP2_Rv	AAACCGGCAGTGAGCCCGGTCT
DreESRP2_genotype_Fw	GCAAACTTCACCGGACTCTA
DreESRP2_genotype_Rv	CTTCCCTCCCTCACTCACTC
DreESRP1_ORF_ATG_Fw	ATGACGGTTAATCCCGACTA
DreESRP1_ORF_STOP_Rv	TTATGAAAGAATATCATTAG
DreESRP2_ORF_ATG_Fw	ATGGCTTCGCACAGTGATAC
DreESRP2_ORF_ATG_Rv	CTACAGACAAAGCCACTCTT
DreEsrp1_qPCR_Fw	GGCACTGCAGAGACACAAGC
DreEsrp1_qPCR_Rv	CGCATGCGGACGATAATCTG
DreEsrp2_qPCR_Fw	GTGAAGGACATGGTTCTGC
DreEsrp2_qPCR_Rv	CACGAGCTCTGATGACCGTC
HsaARHGAP17_AS_Fw	TTTGTACCTCTCACCACCCCG
HsaARHGAP17_AS_Rv	GCAGTGTATGCGGGCTGG
HsaExoc7_AS_Fw1	TACCAGATACGCTCCAGCCAG
HsaExoc7_AS_Rv1	CCACGTCCAGCATGTCATCTC
HsaFGFR1_C1_Fw1	CATCCTGCAAGCAGGGTTGC
HsaFGFR1_IIIb_Rv1	CAGTGAGCCACGCAGACTGG
HsaFGFR1_IIIc_Rv1	TCGGTGGTATTAACTCCAGC
CroESRP_probe_Fw	GCTGCTGAATTGCGTTGTTG
CroESRP_probe_Rv	TTCAGCTCGCATCGTGCTCG
CroTwist-like1_promoter_Fw	ACCACAGCTTCTATTATATATTAACCTC
CroTwist-like1_promoter_Rv	TATCGTGTGTTGATTGATTTGAAAG
CroTwist-like2_probe_Fw	ATGCCAAAATCACCAGTCAAG
CroTwist-like2_probe_Rv	CCACACATTAAGTTTCTGGC
CroESRP_transORF_Fw	ATGACGAGCCCAATCATACCCAC
CroESRP_transORF_Rv	TTAAATAAGCAGCAGTTCGATGTACC
BlaESRP_probe_Fw	CGAGGACTCAACATCACCAA
BlaESRP_probe_Rv	GCCGTTCTTGTTCAGCTGAC

SpuEsrp_probe_Fw	GCAGGCCTCGGTCACCATCA
SpuEsrp_probe_Fw	GCAAAGCACGTGATGCTCCA
SpuEsrp_intron2ret_mRNA_Fw	ACTCAGCATAAGACCTGATG
SpuEsrp_intron2ret_gDNA_Fw	TGGCATGGACTCACTTAAC
SpuEsrp_intron2ret_mRNA_Rv	TTCACTGCCGTATCGTCTGG
DreMeis1b_AS_Fw	AGCAGCACATGGGAATCAGAC
DreMeis1b_AS_Rv	AGGGATGTATGAACGCATGGG
DrePtprfb_AS_Fw	TAATGGATGTCGCTCTGCCAG
DrePtprfb_AS_Rv	CGAGCTCCATCACCTTTGGTT
DreSnap23.1_AS_Fw	CGAAGGAATGTAGGAAAGAGTTTGGA
DreSnap23.1_AS_Rv	CTCATCTGTAACTTGGTTGGCTCT
DreUso1_AS_Fw	GTACAGTTGGCCACCAGTCTG
DreUso1_AS_Rv	GCAGAGCAACATGAGGAGACC
DreSpast_AS_Fw	GATCACCAACCTGTCCATGGC
DreSpast_AS_Rv	GAGGGAGTGCAGTTCAGACCT
DreMark2b_AS_Fw	ACTCTAGGAGTGCAAAACGGC
DreMark2b_AS_Rv	TCTTCGTTGTTGGCTGTTCCC
DreSept7_AS_Fw	GGCATCGACAACAACAAAGCC
DreSept7_AS_Rv	CCTGCTCCATCTCCATCTCCA
DreFlna_AS_Fw	GTACCGTGTGCACACCTGATG
DreFlna_AS_Rv	ACCCAATTGCCCATCTGTAGC
DreLSP1_AS_Fw	GGAGGGATGTGATGGAGAAGC
DreLSP1_AS_Rv	GCGGTTTAAAGACTCGGTCCT
DreMagi1b_AS_Fw	GAGAGCACCAAGGGCATGAAG
DreMagi1b_AS_Rv	AGGAGCTGAGTTCACTTCCGA
DreSfswap_AS_Fw	GTCTCAGTCTCGGTCCCCC
DreSfswap_AS_Rv	CTGAGTGATTTTGCTTTGTACTGA
DreARHGAP12a_AS_Fw	ACACCTCTGATGCCAGGAGAT
DreARHGAP12a_AS_Rv	CGGACTTTCTTTCCATGTTCAGTG
DreVaspb_AS_Fw	AGAAGACCATGGGAAAAATCCGC
DreVaspb_AS_Rv	CTGTTTAATTCGTTCCATCTCTGACT
DreTnc_AS_Fw	CCTGGGATCTGGACAGAGGAG
DreTnc_AS_Rv	ACGGGTATGTGGCATAGTCCA
DreCol4a3bpa_AS_Fw	TCCCAGGTGGAAAGGAGCAG
DreCol4a3bpa_AS_Rv	ACAACCAGTTGCCAGTTAGCA
SpuExoc7_AS_Fw	GGATCTAACGTCTCGGAGAC
SpuExoc7_AS_Rv	CTAGAGCCTTGGAACCTGTT
SpuFam40_AS_Fw	GGTGTATTCAGTGAATGTTC
SpuFam40_AS_Rv	CTGCAGAACTTAGTCACCAT

SpuMyo5_AS_Fw	CATGCGGACATATCTACTGG
SpuMyo5_AS_Rv	CTACCAGATTAGCCTTGTCA
SpuDdc_AS_Fw	AGCAAGTGAATCAACTCTAG
SpuDdc_AS_Rv	GGACCCAGCTCCTTGAGATT
SpuCbl_AS_Fw	CAAGGTCCTCGGTCCAGTAC
SpuCbl_AS_Rv	GTTGATGGACATGATGTGAG
SpuIft88_AS_Fw	AATCTAGAGCAAGATGAGAC
SpuIft88_AS_Rv	CTGCCGCTGTTCCCATGATG
SpuC8orf34_AS_Fw	CTGAGAAAGGACCCACTGTC
SpuC8orf34_AS_Rv	TCAACTGAGCCTGTTCATAC
SpuFam96a_AS_Fw	TAGGTCTTTGTCTGAGAGTC
SpuFam96a_AS_Rv	TCTATGCTGACACAATGTTC
SpuLyrm7_AS_Fw	GGTGCTGTCTCTGTATAAGT
SpuLyrm7_AS_Rv	GTGAATGCCTTGAGCTACAG
SpuKIAA0427_AS_Fw	ACCTGCTACCTGTGCTGCCT
SpuKIAA0427_AS_Rv	CATCCGCTTGCTCTAAGCTC
SpuNup54_AS_Fw	GGTGCCAAGCCAGGAGGTTC
SpuNup54_AS_Rv	CTCATCGCCATAGATGCTGG
SpuSlain2_AS_Fw	CTCGTAGCTCAAGCGTTAGC
SpuSlain2_AS_Rv	CTGAGTCTGTTGACAGCGTT
DreFGFR1a_C1_Fw	GCCTGCAAACCGTACCGCAG
DreFGFR1a_IIIb_Rv	TGTGTGTCCGAGCTGTTGAC
DreFGFR1a_IIIc_Rv	ATAGACAGTCAACCATGCAG
DreFGFR1b_C1_Fw	CAGACCCATCCTGTACGCTG
DreFGFR1b_IIIb_Rv	GGTGTCCGAGCTATTGACGC
DreFGFR1b_IIIc_Rv	TAACAACGGTCAGCCATGCT
DreFGFR2_C1_Fw	CTCCTCACCGGCCCATTCTC
DreFGFR2_IIIb_Rv	CGAGCTGTTTATGCCCGAGC
DreFGFR2_IIIc_Rv	ACCGTCAACCAAGCAGTGTG
DreFGFR3_C1_Fw	TCACCGGCCCATCTTACAGG
DreFGFR3_IIIb_Rv	GTCAGCGTATCTTGGGCCTC
DreFGFR3_IIIc_Rv	TAAGCCAAGCAGAGTGATGG
BlaFGFR_C1_AS_Fw	GTGGACTTCCAGTGTAAGGT
BlaFGFR_IIIb_AS_Rv	AGCACTCTGATTGACGCTTC
BlaFGFR_IIIc_AS_Rv	TGTGTTATTCACATCCGCTG
BlaFGFR_C2_AS_Rv	GTAAGGGTTTCTTCAGCTGG
BlaESRPexp_Fw	CAGCGAACTTTCCGACGAGG
BlaESRPexp_Rv	CGCTGTCCCTGAGGAACGCT
BlaGAPDH_exp_Fw	CCGATGTTCGTCATGGGTGT

BlaGAPDH_exp_Rv	ATCGTTGATGACCTTGGCCAG
BlaFGFR_Mg_Fw	GCGGAAGTACCTCATTGATA
BlaFGFR_Mg_Rv	TAGTGATGAGGACAGTCTTG
BlaFGFR_Mg-ΔIIIx_Fw	GTGGTGACAATGCATTGAGA
BlaFGFR_Mg-ΔIIIx_Rv	ACCACACAAATCTGCGAACC
BlaESRP_ORF_Fw	CGCATCTCGAACTGGTTCGC
BlaESRP_ORF_Rv	TATGGTCTGACATCAATGGC
HsaSLK_AS_Fw	GCTTGCGAGATGAAGCCAAAC
HsaSLK_AS_Rv	GCTAACTCTGCCTTCTGCTGC
HsaOSBPL3_AS_Fw	AAATGAGCCAGCTCCTGCAAA
HsaOSBPL3_AS_Rv	TGAGGTTTCAGAGCCATCAGA
HsaFLNB_AS_Fw	GGCGAAGAAGTAGGCTTTGTGG
HsaFLNB_AS_Rv	GCCGTTCATGTCACTCACTGG
HsaMAGI1_AS_Fw	TCTTGGTTAGACCCTCGGTGC
HsaMAGI1_AS_Rv	AGATACCATAGACAGGGTCTTCA
HsaRALGPS2_AS_Fw	GTAGCAGCGATGGTTCTGAACT
HsaRALGPS2_AS_Rv	GCTTTTTGCCTTCTTTTAACAAAGT
HsaSCRIB_AS_Fw	AGCACTGAGGAGGAGGACAAG
HsaSCRIB_AS_Rv	CCTTATAGGGTGTGGAGCCC

Supplementary Table 3. This table contains statistics for sea urchin, zebrafish, mouse and human RNA-seq data.

Sample ID	Species	Length	Reads	%mRNA	%mRNA_m	%gDNA	%gDNA_m	Accession	Source
Embr_5dpf_ESRP_WT_a	*Danio rerio*	125PE	155,379,946	77.95%	9.11%	60.15%	10.25%	NA	This study
Embr_5dpf_ESRP_WT_b	*Danio rerio*	125PE	139,723,611	77.35%	9.11%	59.33%	10.08%	NA	This study
Embr_5dpf_ESRP_DKO_a	*Danio rerio*	125PE	147,085,624	80.15%	6.17%	60.06%	8.55%	NA	This study
Embr_5dpf_ESRP_DKO_b	*Danio rerio*	125PE	140,076,135	81.20%	6.41%	62.44%	8.30%	NA	This study
Embr_24h_Esrp_Cont_e	*Strongylocentrotus purpuratus*	125PE	77,394,331	27.84%	2.05%	47.96%	15.26%	NA	This study
Embr_24h_Esrp_Cont_f	*Strongylocentrotus purpuratus*	125PE	72,923,440	29.05%	2.93%	49.40%	15.89%	NA	This study
Embr_24h_Esrp_KD_e	*Strongylocentrotus purpuratus*	125PE	82,623,468	25.97%	2.20%	44.53%	15.65%	NA	This study
Embr_24h_Esrp_KD_f	*Strongylocentrotus purpuratus*	125PE	87,361,488	25.42%	2.69%	47.00%	16.15%	NA	This study
E18_Epidermis_Cont_a	*Mus musculus*	100PE	45,927,980	74.32%	8.00%	61.02%	25.42%	SRR1725983	PMID: 26371508
E18_Epidermis_Cont_b	*Mus musculus*	100PE	83,095,958	79.59%	8.19%	62.15%	23.74%	SRR1725984	PMID: 26371508
E18_Epidermis_Esrp12_KO_a	*Mus musculus*	100PE	39,300,846	69.61%	9.50%	56.81%	31.60%	SRR1725976	PMID: 26371508
E18_Epidermis_Esrp12_KO_b	*Mus musculus*	100PE	54,796,272	74.57%	8.92%	59.65%	28.11%	SRR1725977	PMID: 26371508
CL_H358_ESRP_Cont_a	*Homo sapiens*	101PE	41,442,942	74.41%	4.07%	61.55%	21.32%	SRR2966415	PMID:27044866
CL_H358_ESRP_Cont_b	*Homo sapiens*	101PE	67,887,074	74.07%	4.05%	61.36%	21.32%	SRR2966416	PMID:27044866
CL_H358_ESRP_Cont_c	*Homo sapiens*	101PE	67,933,853	74.24%	4.09%	61.29%	21.47%	SRR2966417	PMID:27044866
CL_H358_ESRP_KD_a	*Homo sapiens*	101PE	40,653,351	74.82%	4.13%	61.08%	21.97%	SRR2966412	PMID:27044866
CL_H358_ESRP_KD_b	*Homo sapiens*	101PE	57,451,860	74.83%	4.12%	61.19%	22.07%	SRR2966413	PMID:27044866
CL_H358_ESRP_KD_c	*Homo sapiens*	101PE	51,094,649	74.07%	4.06%	61.92%	21.77%	SRR2966414	PMID:27044866
CL_PNT2_ESRP_KD	*Homo sapiens*	76SE	74,468,344	70.33%	3.27%	41.28%	15.75%	SRR436883	PMID:22354987
CL_PNT2_ESRP_Cont	*Homo sapiens*	76SE	59,124,618	70.70%	3.34%	43.98%	15.34%	SRR436884	PMID:22354987
CL_MB231_ESRP_Cont	*Homo sapiens*	76SE	136,249,697	72.32%	3.92%	37.76%	16.48%	SRR436885	PMID:22354987
CL_MB231_ESRP_OV	*Homo sapiens*	76SE	120,901,583	74.63%	4.61%	45.73%	19.49%	SRR436886	PMID:22354987

Supplementary Table 4. It contains the translated protein sequences for *Esrp* putative orthologous from Choanoflagellates used for Supplementary Fig. 2.

Species, ID, type	Translated sequence
Salpingoeca_urceolata_ATCC_50560<br>m.46455, g.46455<br>type:internal, len:599 (-)<br>comp11862_c1_seq1	ADDAAAAPVADPSATEVASNAAPATEAVSAAPVGAVANGQV<br>VPELPAKDYDYLVVIDLESSCPEEDIVESVAEVIIFAWLVVDA<br>ASGTISYEHGTTHVRPERLPLSAYTTRVTGVESAELEEAKPL<br>AATIAELQEYLESQITSQGKSYCLVTDGQWDVRQGLAPEAE<br>RKGITLPAELQTFFDLKKQYTAFTKHEGATPTLAVMAAALD<br>NVALESGEHNNGIVECRNIAKIVCELINRGHKFQVPEMVSAY<br>SKTVAGAEAAENAASVTGSNTDTVVQLRGLPWQATEVDVV<br>EFFSGLNIVPGGVVMCLGPQGRPNGEAFVQFTDGQQRDLAL<br>GRHKQLMQSRYIEVYESSQVEFDGAKQALSSSDSDTFIIRMR<br>GLPYSATAEDITSFFAPIPLADNGIYIVQTPLGRPAGEAFAQFP<br>TLEDMRSGMERHRQMMQSRYIELFKSSLEEVHRVVEATTRQ<br>AQQAAANEQAIAAGAQASGQFVVRLRGLPFSATVPEIMEFL<br>EGVEIETNGVQIALNASGRPSGEAYVQVCSFPDMQKALQHD<br>RKHMGPRYVEVFSCSVQQMMAGLDPRSGTMRSNRGGYGG<br>GNQYGGGYGGGQAGGYGGGQ
Salpingoeca_qvevrii_ATCC_50929<br>m.85620, g.85620<br>type:internal len:651 (+)<br>comp8395_c2_seq1	SEEAAAATTAAVPAEEASSAAAPEAVSAPAEAPPAAPAAAEE<br>SAEGAETEASAAPVAEAAPAEEEVDQSVDVLVILDLESTVDE<br>MEEKPSEILVFAWVFLDTATGEILHEQLLYVKPSHMEQLTAY<br>CRKVTGVTQENLNDASELVDAVDWLKNNINMNLINKQKSF<br>SFMTDGPWDLRQCLVHECIDKKIELESYFLAYHDIRKEFASC<br>HPEHGLPEPLTLKKMAEFLDITLERHEFHNGISECKNIAKIAA<br>ALLKSGHKLKKREQIRMYGVQAMPPSGATAGDAPGDAASG<br>DAAIGTAGVPHPVAEAQPFSGNRDDCCALLRGLPWQATEKD<br>VLEFFTGLNVVKDGVIFKYSYQGRPSGEAYVEFSNPEQRLLA<br>LERHRQLLGERYIEVFKTTHHELESARSQAPQSSGDGASYIV<br>RMRGLPYSTTPEDVQAFFSPIPLVDDGLYIVKMPDGRLTGEA<br>YAQFHSEEDMHKGLERHKQMLGSRYVELFPSSVEEAKRVSS<br>QMSATPMVPSHHAAGQSDGVVRLRGLPYSATEMDITNFFQG<br>LSVAEVHITLNMDRRPSGEALVRFSNPQDVQGALKFHRNM<br>MGTRYIEVFRSDFHEWNNVTQGGGYGGGYGGGYGGQGGY<br>GGGYGGGYGGGYGGGYGQGYGGYGGQQGGYGQ
Mylnosiga_fluctuans_ATCC_50635<br>m.111042, g.111042<br>type:5prime_partial len:716 (+)<br>comp9077_c0_seq1	RTYRAVFSYNMAAVRELLVLDLETNGAEIPETTQMSWLVFDI<br>ASQMEQNEHSTFVHPAANGAGTAYWQHCTGISEADIQGGRS<br>FAEAVTELGTYVNSRDPGTVALCTDGPWDVRQCLLPEAQRK<br>GISLDTFFSTYYDVREEFGACVDGPRAVSLVDLHHRLGLRGT

	EWGSRVGLPECHRIVAVLKELLKRGATLSRPQTVQAAAQGT LTDAACAVRLRNVAWKAGVADIKAFFCGLDIVQGGVALSFD QMGRPSGEAVVMFTSSDMAALACDRSHQILTDRVVEVNPAV EEDLGILRGGRSSHVVRLRGLPYSATRDNIKDFFSPISLEADSI VIAQNASGQPSGEAFVEFTSEGDVASAMRCNGQHMGSRFIE LFTSSPEERARATRFQERRSEPRPAQPSRPAEGHVMLRSLPW TVREAEIIAFFKGLDVLPDGVQLLMLPDGRPSGGAIVAFGTA ADAESALLLSKSRLGTRVIEIVPCEAPPPPLAGPSGSGYAEAP RDPYSYRDRYERSAYDDPYAYGRGYDGPSRGYSRYPPQHYP SSRGGPYASSYARGASSSYAPDDYYAADYGQAGYEAAGSA DTAYASGGYSDTQASYTGGYEGYSSAYARQSSYAGEYGSQY DPAAYPRSSAPRGAYAPPASGATLRMRGLPFSAGAEDVLAFF SDYRVVPDSLSGPSNGQAIISFETPEEAQRAMQERQRQYMG TRFIELFAA*

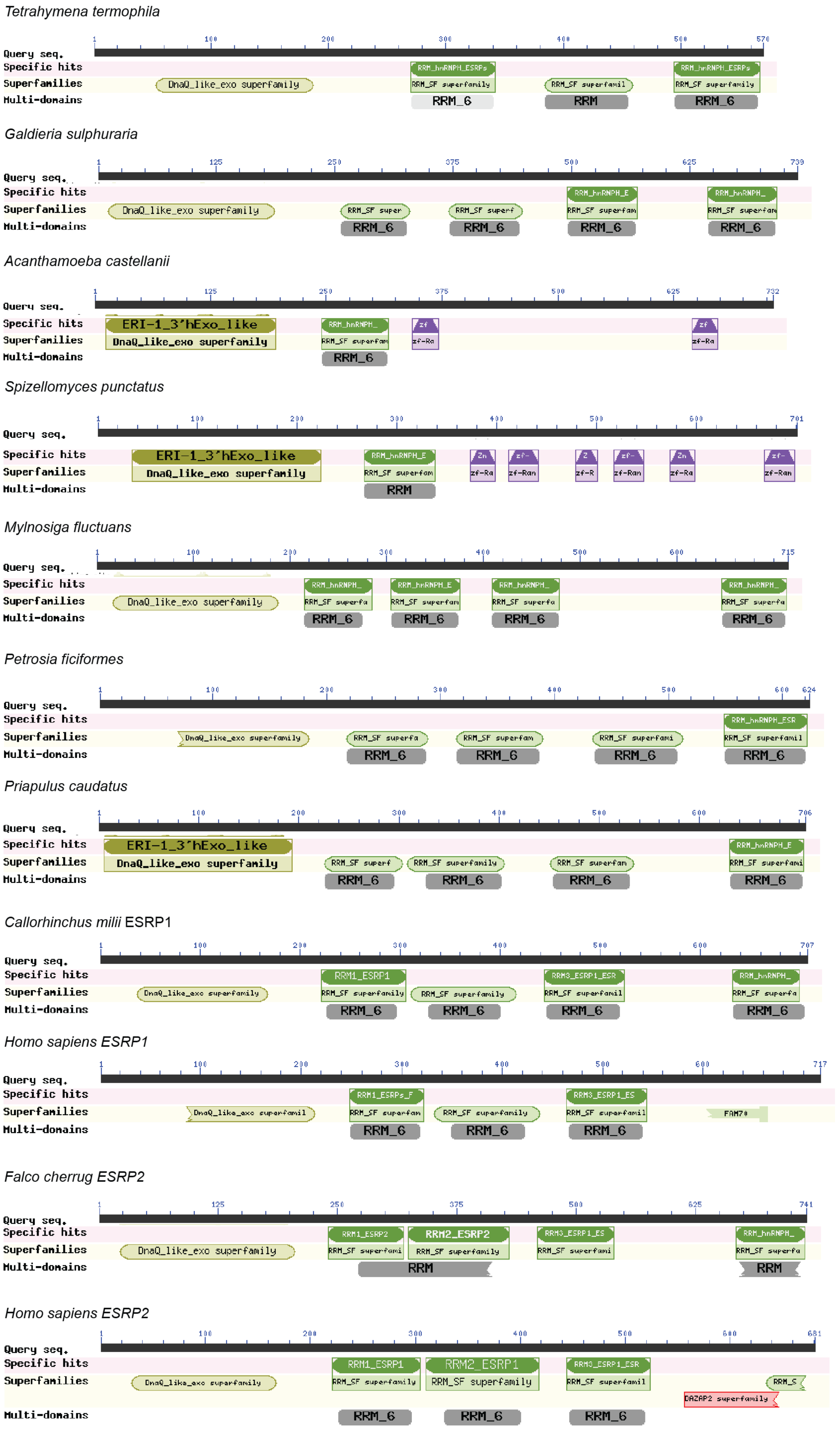

Tetrahymena termophila
Query seq.
Specific hits
Superfamilies
Multi-domains
DnaQ_like_exo superfamily
RRM_6
RRM
Galdieria sulphuraria
Acanthamoeba castellanii
ERI-1_3'hExo_like
DnaQ_like_exo superfamily
Spizellomyces punctatus
Mylnosiga fluctuans
Petrosia ficiformes
Priapulus caudatus
Callorhinchus milii ESRP1
RRM1_ESRP1
Homo sapiens ESRP1
Falco cherrug ESRP2
RRM2_ESRP2
Homo sapiens ESRP2
RRM1_ESRP1
RRM2_ESRP1
RRM_SF superfamily
DAZAP2 superfamily

Supplementary Figure 1 | Protein domain composition of *Esrp* homologous genes in eukaryotic species.

All represented eukaryotes share a DnaQ-like exonuclease domain in their *Esrp* homologous genes. Variation arises from the number of RRM6 domains, a specific type of RNA-binding domain. The alveolata species *Tetrahymena termophila*, and the rodhophyte *Galdieria sulphuraria* have three and four RRM6 domains, respectively. In the amebozoan *Acanthamoeba castellanii* and the fungi *Spizellomyces punctatus*, more closely related to metazoans, only one RRM6 domain was found. Zinc finger domains were also detected in those species. Four RRM6 domains were found in orthologs from choanoflagelate and metazoan species. However, partial or no detection of a fourth RRM6 domain was observed in some species, especially in *Esrp1* genes from vertebrates. Partial DAZAP2 and FAM70 domains were also detected in human genes, but with low e-values. Other species in the figure are the choanoflagelate *Mylnosiga fluctuans,* the demosponge *Petrosia ficiformis*, the priapulid *Priapulus caudatus*, the chondrichthyan *Callorhincus milii*, the dinosaur *Falco cherrug* and the mammal *Homo sapiens*.

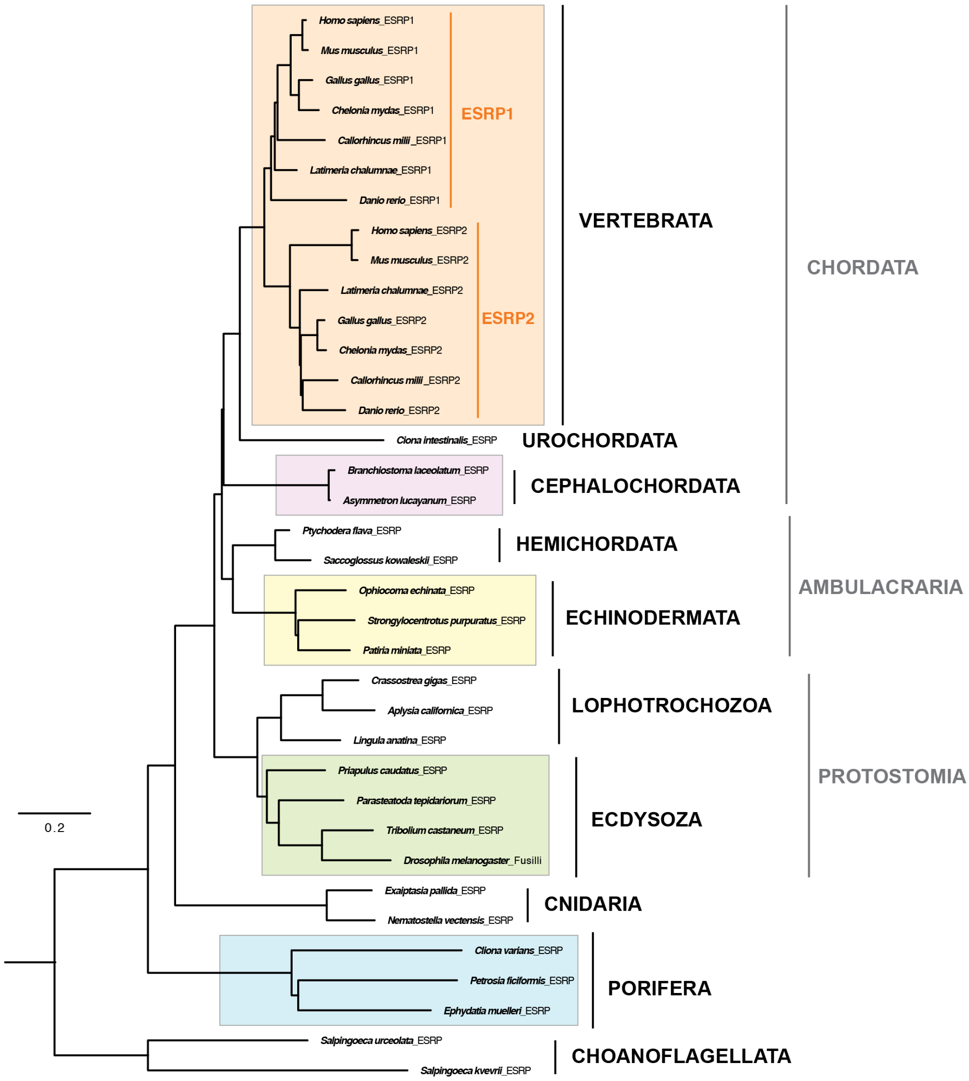

Supplementary Figure 2 | Phylogenetic analysis reveals a lineage-specific duplication of *Esrp* genes in vertebrates.

Most species shown in the tree belong to metazoan lineages, with the exception of choanoflagelates *Salpingoeca urceolata* and *Salpingoeca qvevrii*. Vertebrates: *Homo sapiens* (Hsa), *Mus musculus* (Mmu), *Gallus Gallus* (Gga), *Chelonia mydas* (Cmy), *Callorhincus milii* (Cmi), *Latimeria chalumnae* (Lch) and *Danio rerio* (Dre). Urochordates: *Ciona intestinalis* (Cin). Cephalochordates: *Branchiostoma lanceolatum* (Bla) and *Asymmetron lucayanum* (Alu). Hemichordates: *Ptycodera flava* (Pfl) and *Saccoglossus kowalevskii* (Sko). Echinoderms: *Ophiocoma echinata* (Oec), *Strongylocentrotus purpuratus* (Spu) and *Patiria miniata* (Pmi). Lophotrochozoans: *Crassostea gigas* (Cgi), *Aplysia californica* (Aca) and *Lingula anatina* (Lan). Ecdysozoans: *Priapulus caudatus* (Pca), Parasteatoda tepidariorum (Pte), Tribolium castaneum (Tca) and Drosophila melanogaster (Dre). Cnidarians: *Exaiptasia pallida (Epa) and Nematostella vectensis (Nve). Sponges: Cliona varians (Cva), Petrosia ficiformis (Pfi) and Ephydatia muelleri (Emu).*

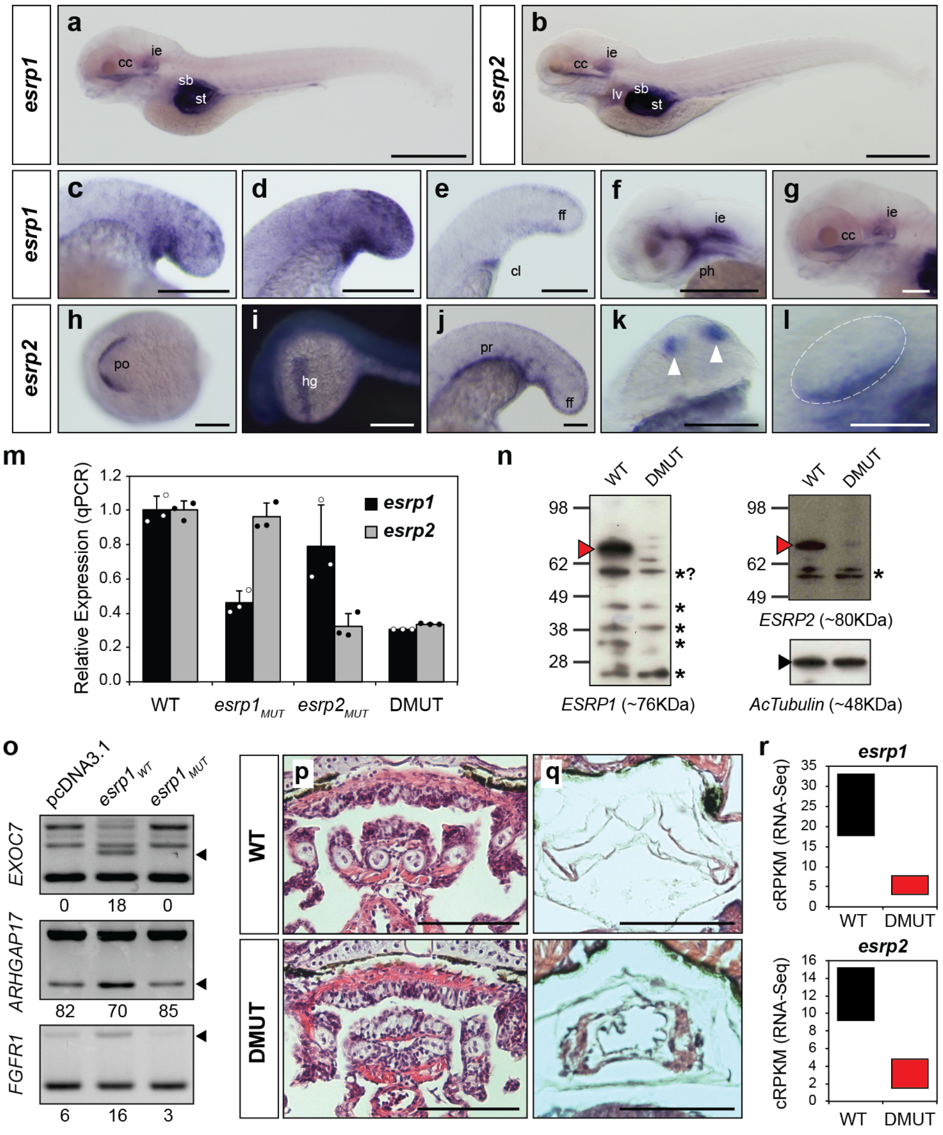

Supplementary Figure 3 | Developmental expression and impact of *Esrp* genes in zebrafish.

(a-l) Additional details of WMISH for *esrp1* and *esrp2* in zebrafish embryos. **(a,b)** Similar expression of *esrp1* and *esrp2* was detected in 5dpf embryos in inner ear (ie) and craniofacial cartilaginous (cc) tissue. Signal was also observed in stomach (st) and swim bladder (sb), although non-specific staining of those hollow organs by NBT/BCIP precipitation could not be ruled out. *esrp2* was additionally found in the liver (lv). **(c-e)** Broad expression of *esrp1* in the tail bud ectoderm was maintained at 18hpf (c), reached its highest intensity by 20hpf (d), and became restricted to the median fin fold (ff) by 24hpf (e), when it was also observed in cloaca (cl) tissue. **(f)** 48hpf embryo heads showed expression of *esrp1* predominantly in pharynx (ph) and inner ear (ie). **(g)** Detail of *esrp1* expression in the head of 5dpf embryos in craniofacial cartilage (cc) and inner ear (ie). **(h-l)** *esrp2* expression was also detected in a few territories where *esrp1* was not observed, including the polster (po) at 14hpf (h), that later develops into the hatching gland (hg) by 24hpf (i), and in the pronephros (pr) (j); it shared expression with *esrp1* in structures such as olfactory placode (white arrowheads) (k, 24hpf) and inner

ear ventral epithelium (l, 32hpf). (**m**) Quantitative PCR (qPCR) assays showing the levels of expression of *esrp1* and *esrp2* genes in pools of 5dpf embryos with different genotypes (wild type [WT], *esrp1* and *esrp2* single mutants and double mutants [DMUT]). Error bars correspond to standard deviations of three technical replicates. **(n)** Western blot assays showing detection of ESRP1 and ESRP2 full-length proteins (red arrow heads) in 5dpf in WT, but not DMUT, embryos. Asterisks mark shorter bands that most likely correspond to unspecific signal based on size. **(o)** Ectopic expression of WT, but not mutant, zebrafish *esrp1* transcripts in human 293T cells produced splicing changes in endogenous *ESRP1* human targets. Black arrowheads indicate *Esrp*-enhanced isoforms. (**p,q**) Phenotypic comparison of transversal sections stained with hematoxylin and eosin from 6dpf embryos shows impaired development of visceral skeleton **(p)** and swim bladder **(q)**. **(r)** Expression of *esrp1* and *esrp2* in 5dpf embryos quantified by RNA-seq using the cRPKM metric shows a clear reduction in the steady-state mRNA levels of both genes in DMUT embryos. Boxes represent the range of expression in the two replicates. Scale bars: 2 mm (a, b, i), 500 µm (d-h, j, k), 250 µm (l), 100 µm (p,q).

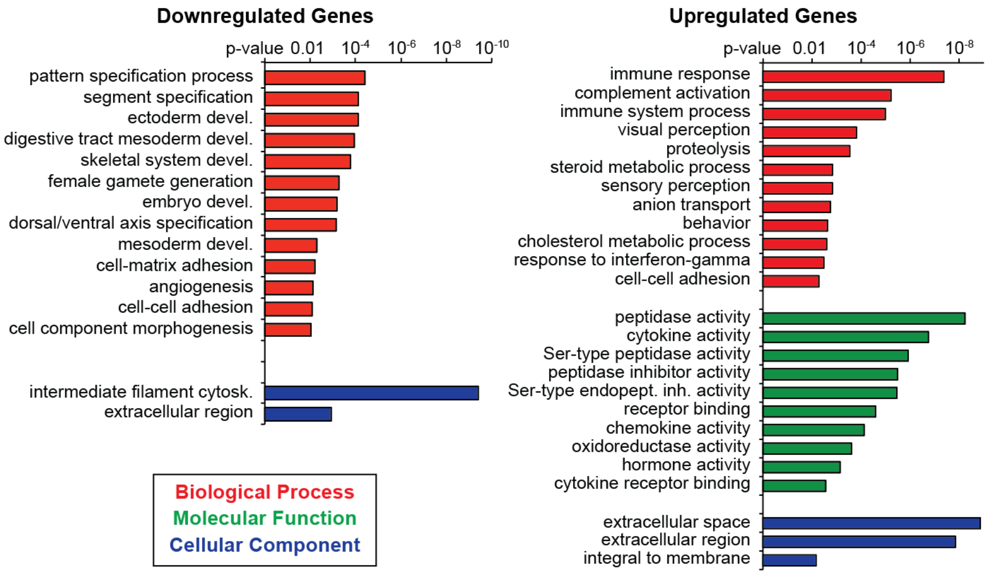

Supplementary Figure 4 | Significantly enriched GO terms for differentially expressed genes in zebrafish

GO enrichment analyses performed using PANTHER for the 248 and 609 downregulated and upregulated genes in zebrafish.

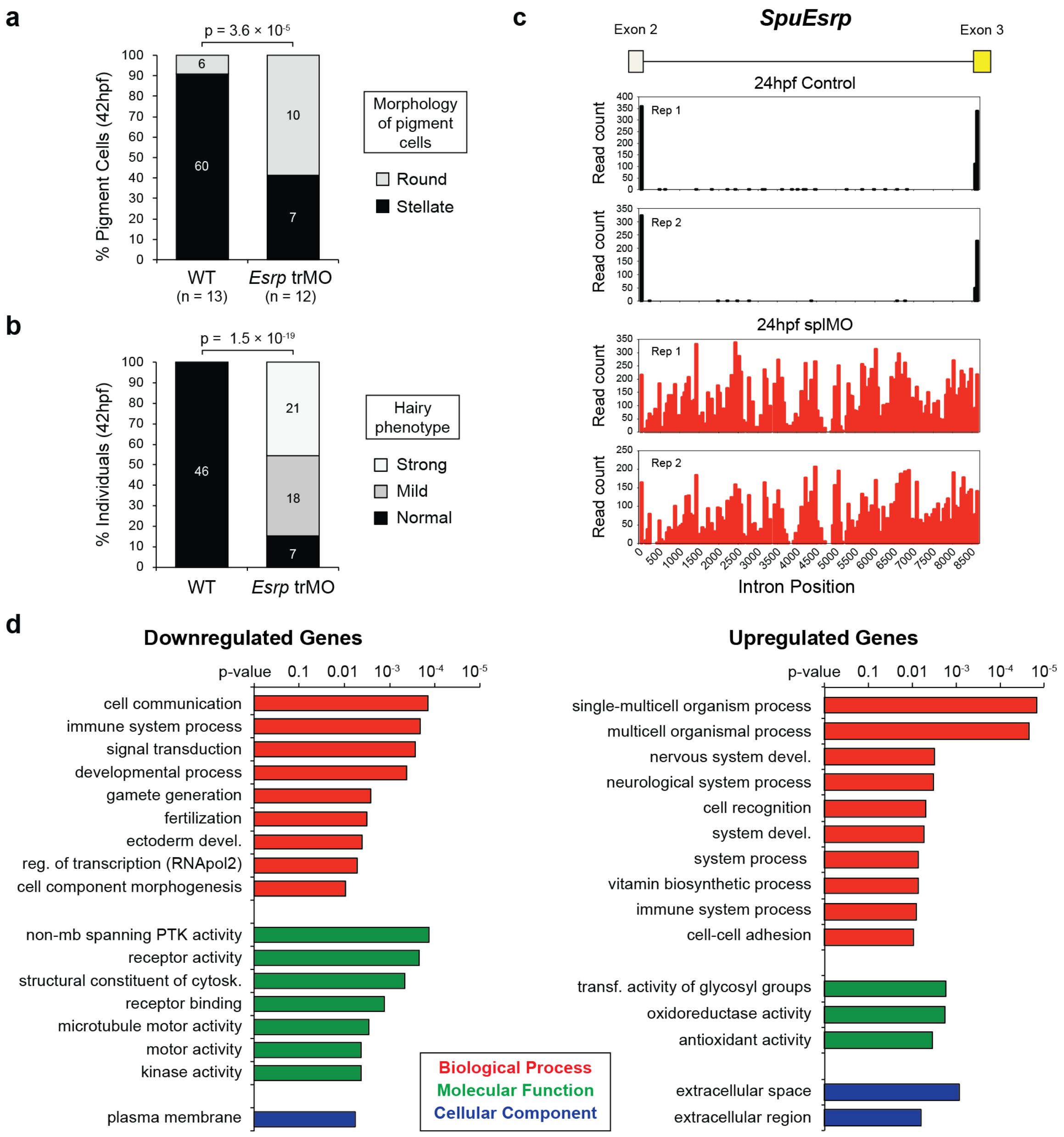

Supplementary Figure 5 | Developmental and transcriptomic impact of *Esrp* in sea urchin

(a) Quantification of pigment cell morphology in control and *Esrp* trMO knockdown embryos at 42hpf. **(b)** Quantification of the 'hairy' phenotype in control and *Esrp* trMO knockdown embryos at 42hpf. Values correspond to the sum of three (a) or two (b) independent experiments. P-values correspond to 2-way (a) or 3-way (b) two-sided Fisher Exact tests. **(c)** RNA-seq reads mapping to the genomic region spanning exons 2 and 3 of *Esrp* in control and SplMO injected embryos. **(d)** GO terms enriched in genes with downregulated and upregulated expression in 24hpf embryos upon splMO treatment. GO analyses was performed using PANTHER for the 580 and 290 downregulated and upregulated genes, respectively, that could be converted to SPU gene identifiers.

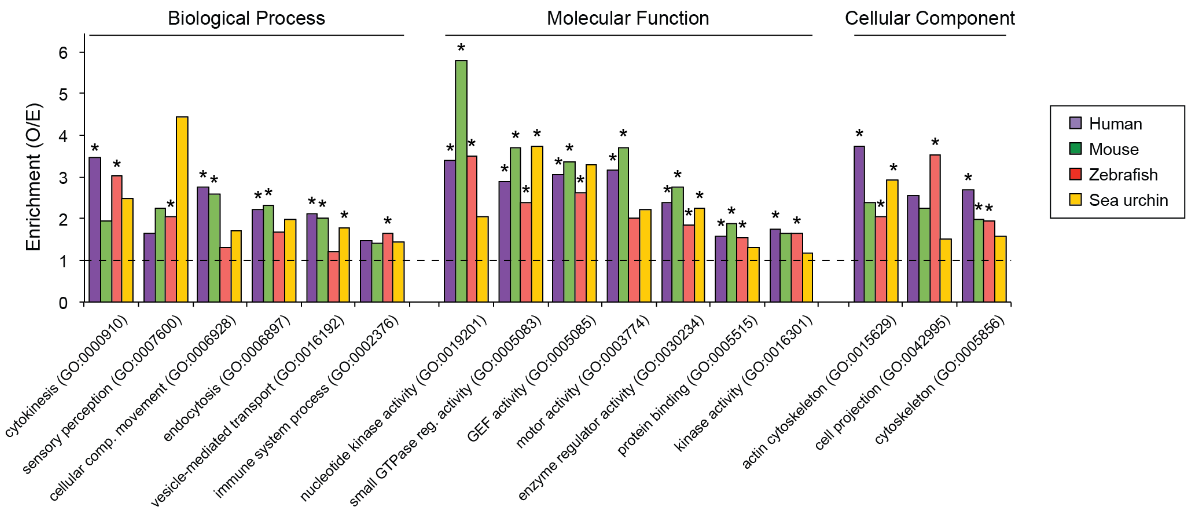

Supplementary Figure 6 | GO enrichment of genes containing *Esrp*-dependent exons among all studied species

GO Categories that were significantly overrepresented in at least one species and showed an observed vs. expected enrichment ratio higher than 1.3 for all species. Asterisks indicate significant overrepresentation ($p<0.05$) in a given species, as calculated by PANTHER.

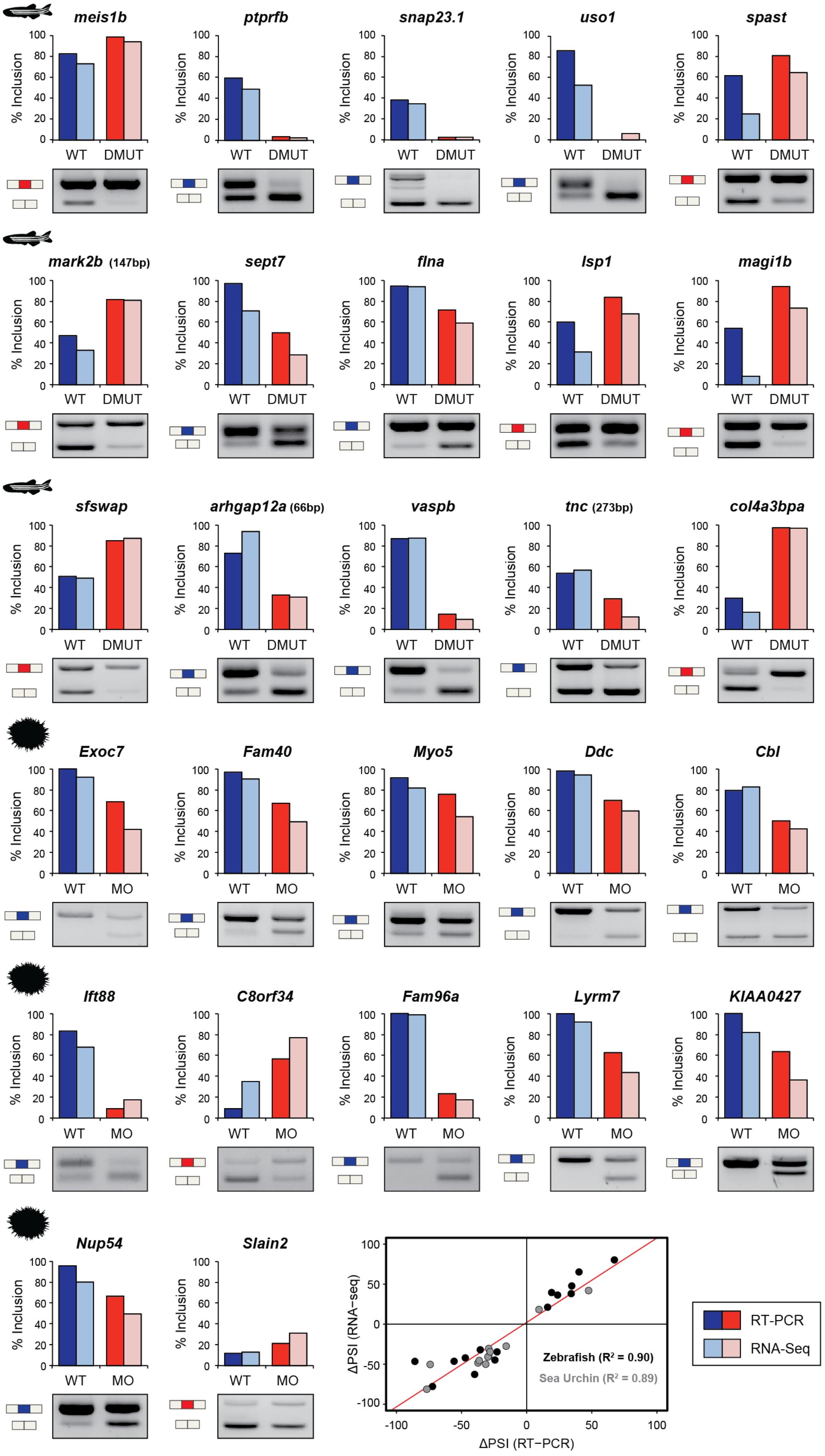
meis1b
ptprfb
snap23.1
uso1
spast
mark2b (147bp)
sept7
flna
lsp1
magi1b
sfswap
arhgap12a (66bp)
vaspb
tnc (273bp)
col4a3bpa
Exoc7
Fam40
Myo5
Ddc
Cbl
Ift88
C8orf34
Fam96a
Lyrm7
KIAA0427
Nup54
Slain2
% Inclusion
0
20
40
60
80
100
WT
DMUT
MO
ΔPSI (RNA-seq)
ΔPSI (RT−PCR)
-100
-50
0
50
100
Zebrafish (R² = 0.90)
Sea Urchin (R² = 0.89)
RT-PCR
RNA-Seq

Supplementary Figure 7 | Validation of *Esrp*-dependent exons by RT-PCR

RT-PCR (dark colors) and RNA-seq (light colors) based quantification of exon inclusion for a subset of 15 and 12 representative *Esrp*-dependent exons in WT (blue) and *Esrp*-depleted (red) zebrafish and sea urchin embryos, respectively. Bottom-right: correlation between ΔPSI values obtained by RNA-seq and PCRs. Strong correlations are observed both in case of zebrafish (R^2=0.90, black dots) and sea urchin (R^2=0.89, grey dots).

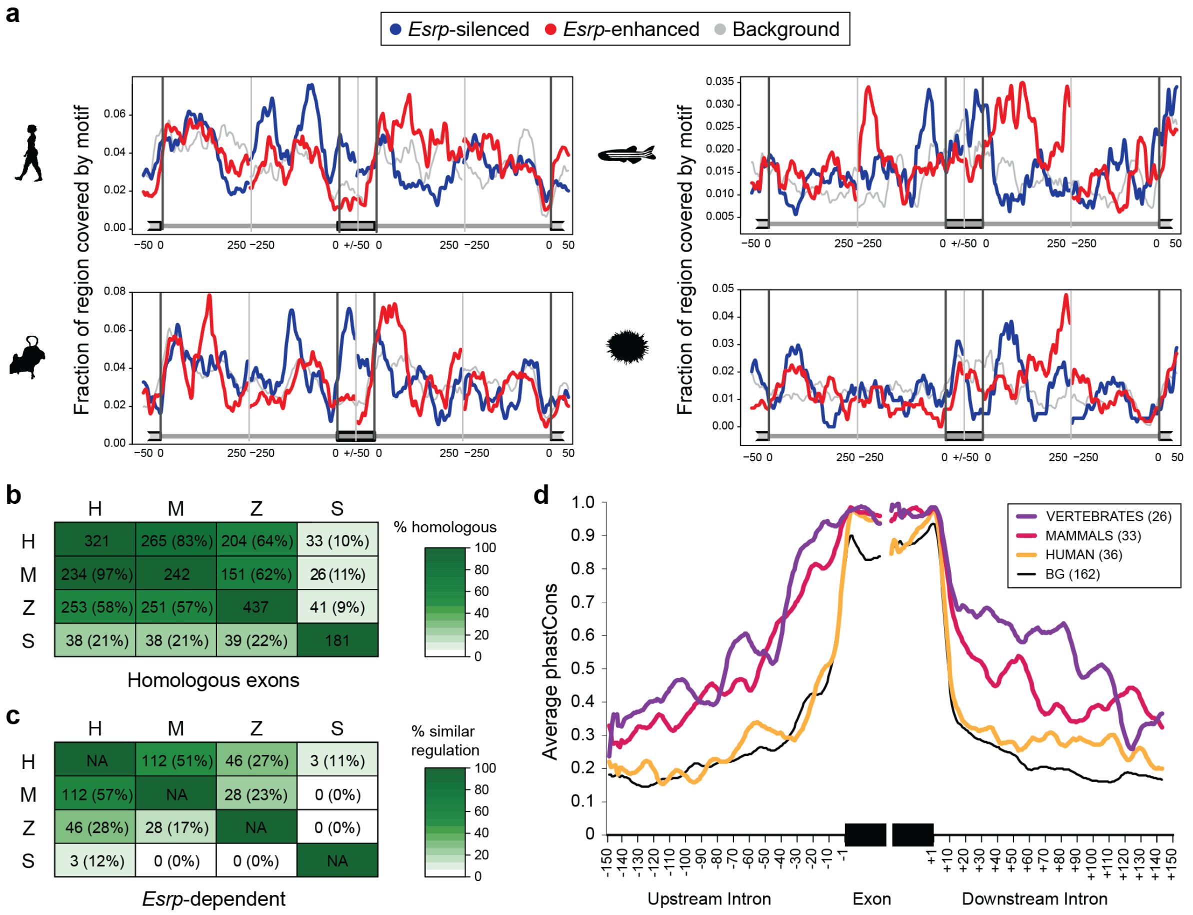

Supplementary Figure 8 | Comparative analyses of *Esrp*-dependent exons

(a) RNA binding map for the top twelve *Esrp* bound 6-mer motifs identified by SELEX-seq. Following the approach described in [15], the fraction of region covered by the motifs is shown for *Esrp*-enhanced (red), *Esrp*-silenced (blue) and non-regulated exons (grey). A sliding window of 51 nt around the plotted position was used to smoothen the signal. **(b)** Pairwise comparison of the percentage of *Esrp*-dependent exons in each species that have a detected homolog in another species. **(c)** Pairwise comparison of the percentage of *Esrp*-dependent exons in each species whose homologs with sufficient read coverage in another species are also *Esrp*-dependent in the same manner (enhanced/repressed). **(d)** Average placental phastCons score in the neighboring intronic sequences of human *Esrp*-dependent exons with homologs in mouse and zebrafish that have shared regulation among the three vertebrate species (purple, 26 exons), between the two mammals (magenta, 33 exons) or only in humans (yellow, 36 exons). As a reference, non-Esrp-dependent exons with homologs in mouse were used (black, 162 exons). A sliding window of 10 nt was used to smoothen the signal by averaging the values for 10 consecutive positions.

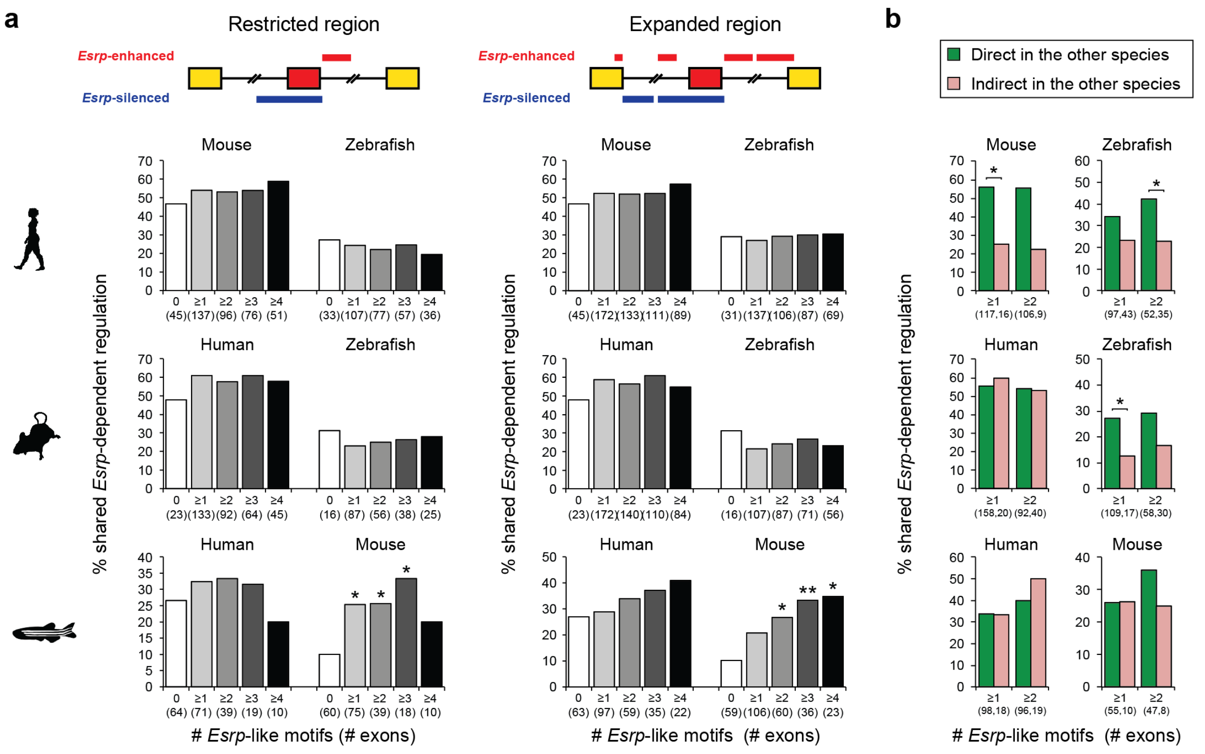

Supplementary Figure 9 | Association between regulatory conservation and presence of Esrp-like motifs

(a) Percentage of *Esrp*-dependent exons with 0, ≥1, ≥2, ≥3 or ≥4 *Esrp*-like motifs in expected positions that have shared regulation in another species. Two scenarios were used to define expected positions based on the type of regulation (see schemes above). Restricted region: for *Esrp*-silenced exons (blue), motifs must be located in the upstream intronic region (up to 250 nt) or in the exon, and, for *Esrp*-enhanced exons (red), in the downstream intronic region, excluding the first 6 nt (the 5′ splice site). Expanded region: in addition to those of the restricted region, we considered, for *Esrp*-silenced exons, the first 250 nt of the upstream intron and, for enhanced exons, the last 250 nt of the downstream intron, the upstream exon and the neighboring upstream intronic sequence excluding the last 50nt. **(b)** Percentage of *Esrp*-dependent exons with *Esrp*-like motifs in expected positions that have shared regulation in another species when the orthologous exon has (green) or does not have (pink) motifs in the equivalent positions for two different thresholds of *Esrp*-like motifs (≥1 or ≥2). Only expanded regions were considered in this analysis. Significant comparisons are indicated with * (0.01<p<0.05) or ** (p<0.01). P-values correspond to 1-tailed Fisher Exact tests of direct versus indirect exons and were not corrected for multiple testing.

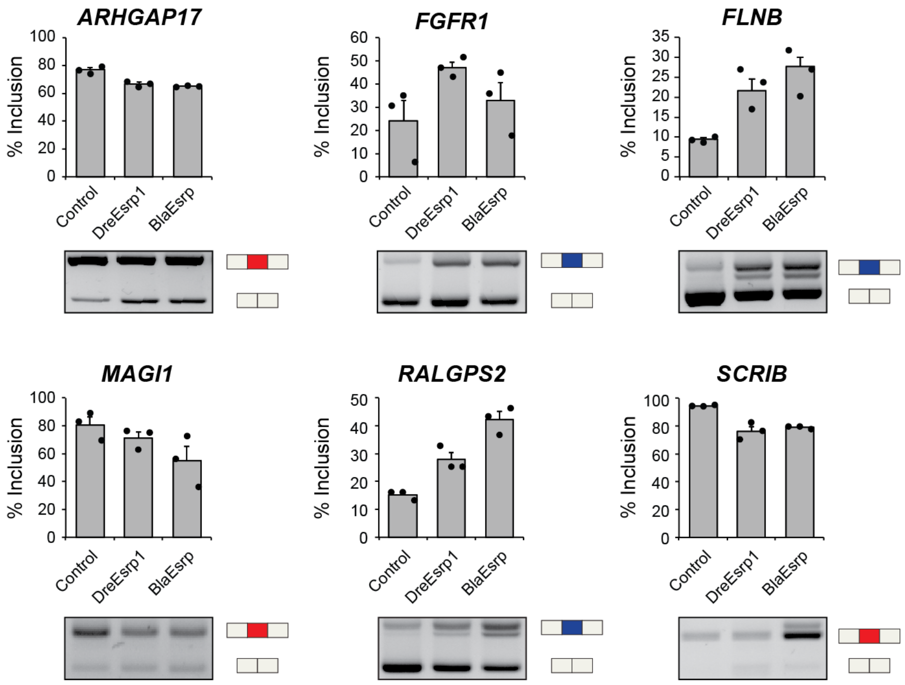

Supplementary Figure 10 | *Esrp* transcripts from amphioxus and zebrafish change the splicing pattern of endogenous human *Esrp*-dependent exons in 293T cells

RT-PCR quantifications of ΔPSI changes in endogenous exons in 293T cells after transfection of expression vectors containing amphioxus (BlaEsrp) or zebrafish (DreEsrp1) ORF sequences. Error bars correspond to the standard error of three independent replicates.

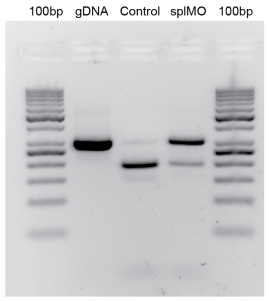

Fig. 4p

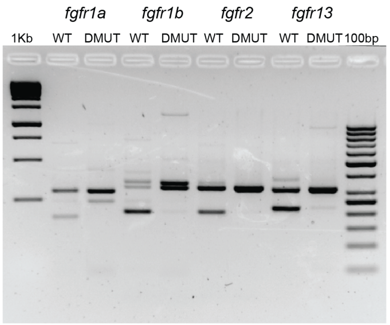

Fig. 6a

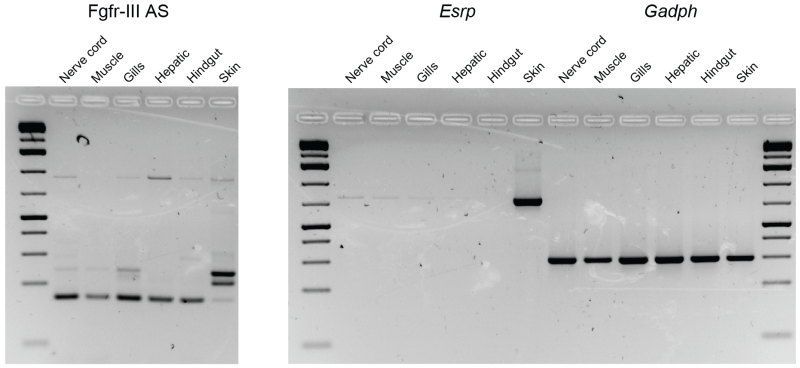

Fig. 6b

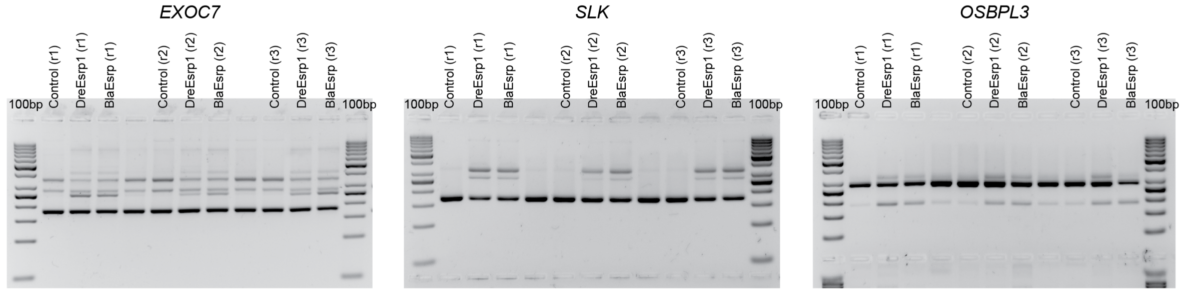

Fig. 6d

Supplementary Figure 11 (part 1/3)

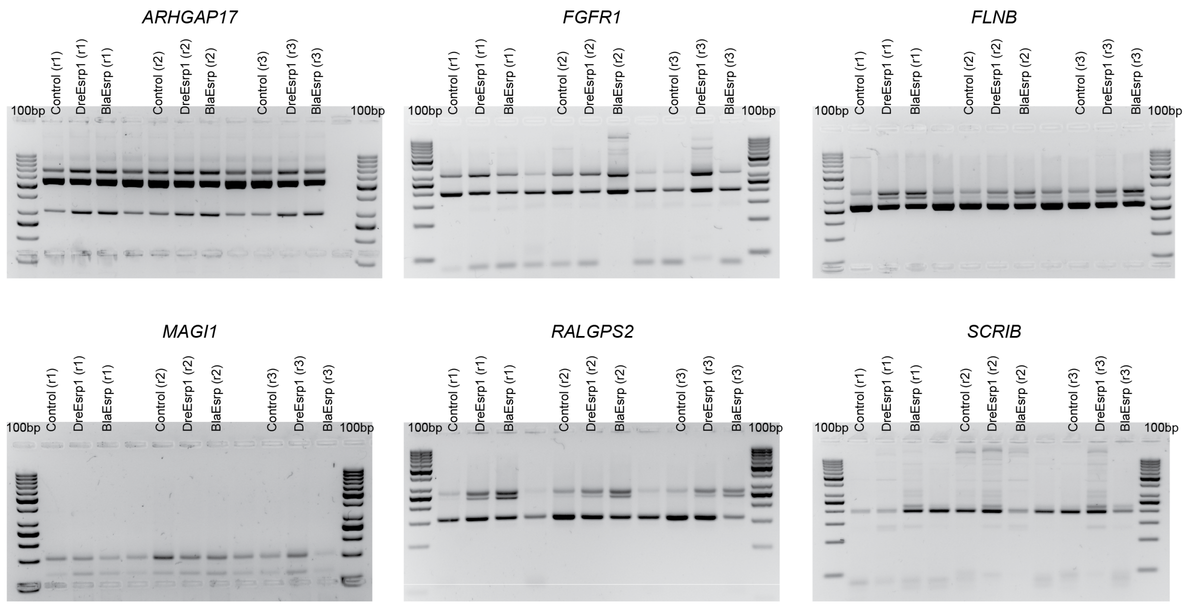

Sup. Fig. 10

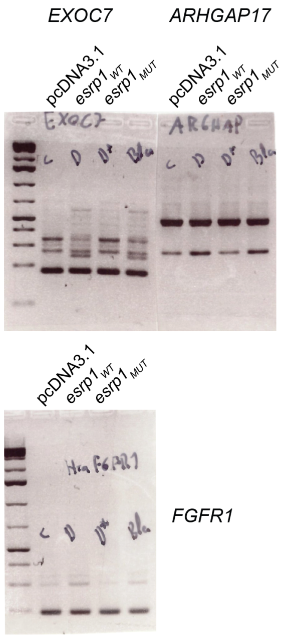

Sup. Fig. 3o

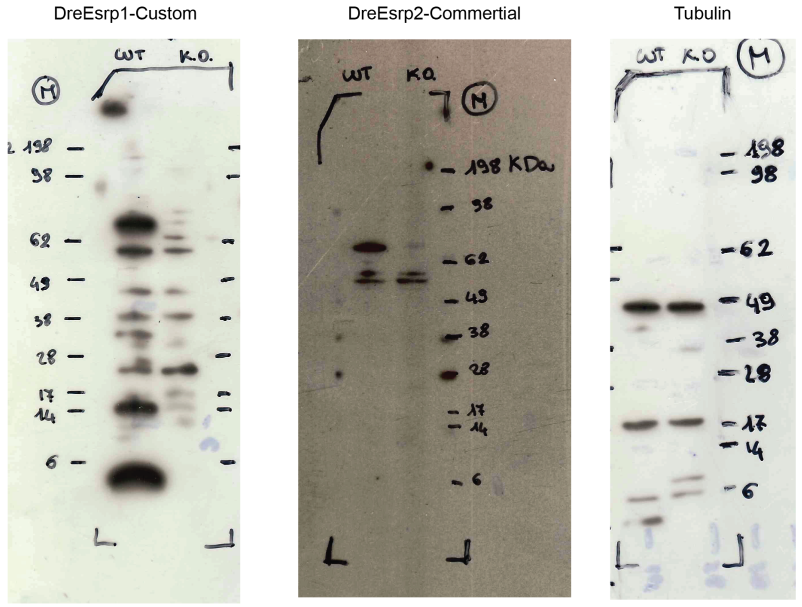

Sup. Fig. 3n

Supplementary Figure 11 (part 2/3)

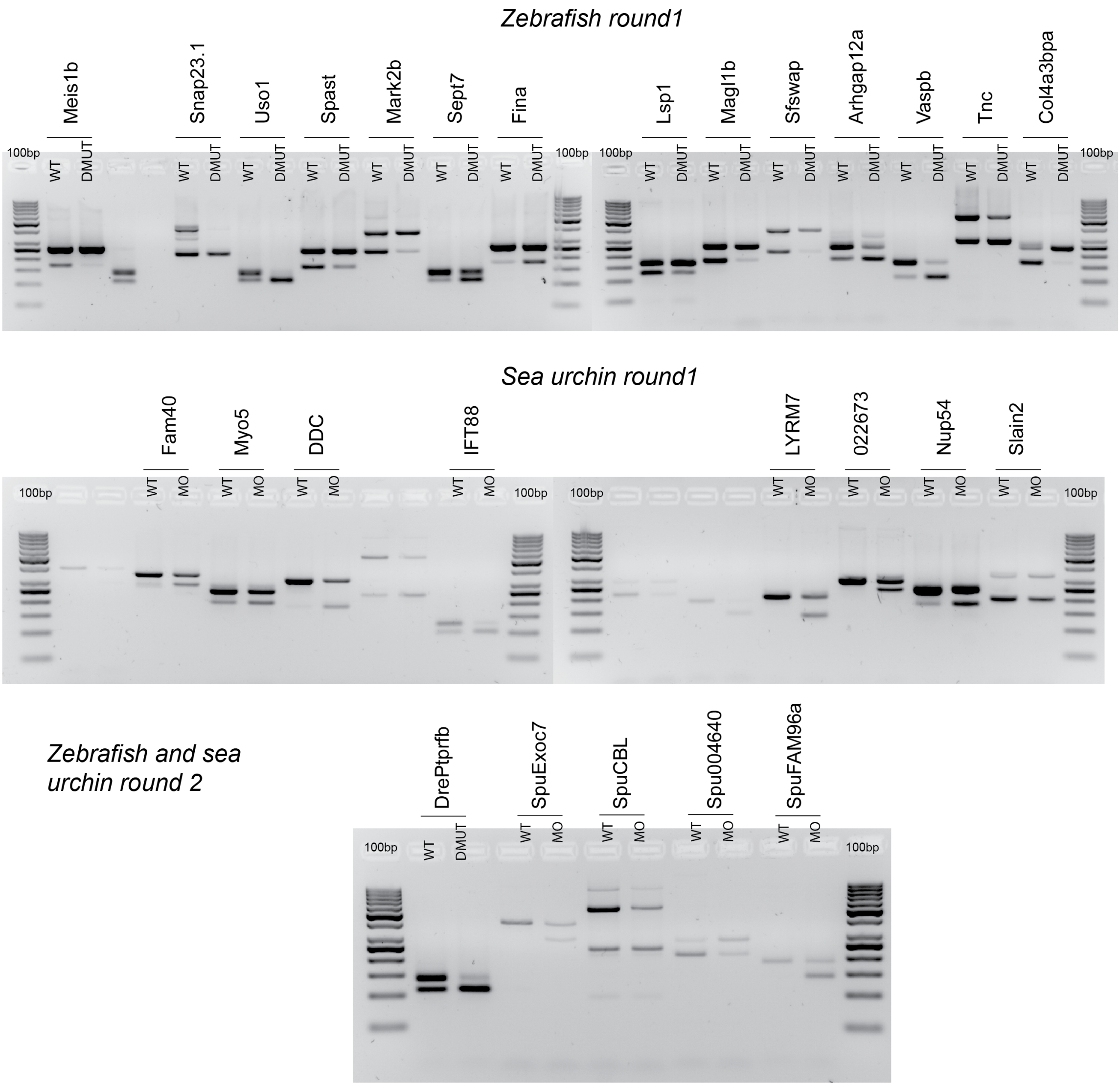

Sup. Fig. 7

Supplementary Figure 11 (part 3/3)

Supplementary Figure 11 | Complete photos of RT-PCR gels and Western blots for all figures.
