# Supplementary material for: Evolutionary recruitment of flexible Esrp-dependent splicing programs into diverse embryonic morphogenetic processes
Source: Nat Commun. 2017 Nov 27;8:1799. doi: 10.1038/s41467-017-01961-y (PMC5703972; doi:10.1038/s41467-017-01961-y)
Supplement: Supplementary file 3 — Description of Additional Supplementary Files [file 41467_2017_1961_MOESM3_ESM.pdf]

Description of Additional Supplementary Files

File Name: Supplementary Data 1
Description: This file contains all AS events of all types detected to be *Esrp*-dependent in sea urchin, zebrafish, mouse and human species.

File Name: Supplementary Data 2
Description: This file contains clusters of identified homologous exons to those detected as *Esrp*-dependent in sea urchin, zebrafish, mouse or human.

File Name: Supplementary Data 3
Description: This table contains the list of differentially expressed genes in WT vs. *Esrp* DMUT zebrafish larva at 5dpf stage.

File Name: Supplementary Data 4
Description: This table contains the list of differentially expressed genes in Control vs. splESRP morpholino injected embryos at 24hpf.
